# Bed net ownership, use and perceptions among women seeking antenatal care in Kinshasa, Democratic Republic of the Congo (DRC): Opportunities for improved maternal and child health

**DOI:** 10.1186/1471-2458-8-331

**Published:** 2008-09-24

**Authors:** Audrey Pettifor, Eboni Taylor, David Nku, Sandra Duvall, Martine Tabala, Steve Meshnick, Frieda Behets

**Affiliations:** 1 Department of Epidemiology, University of North Carolina, Chapel Hill, USA; 2 Salvation Army, Kinshasa, Democratic Republic of the Congo; 3 School of Public Health, Kinshasa, Democratic Republic of the Congo

## Abstract

**Background:**

To describe malaria knowledge, attitudes toward malaria and bed net use, levels of ownership and use of bed nets, and factors associated with ownership and use among pregnant women attending their first antenatal care (ANC) visit in Kinshasa, DRC.

**Methods:**

Women attending their first ANC visit at one maternity in Kinshasa were recruited to take part in a study where they were given free insecticide treated bed nets (ITNs) and then followed up at delivery and 6 months post delivery to assess ITN use. This study describes the baseline levels of bed net ownership and use, attitudes towards net use and factors associated with net use

**Results:**

Among 351 women interviewed at baseline, 115 (33%) already owned a bed net and 86 (25%) reported to have slept under the net the previous night. Cost was reported as the reason for not owning a net by 48% of the 236 women who did not own one. In multivariable analyses, women who had secondary school or higher education were 3.4 times more likely to own a net (95% CI 1.6–7.3) and 2.8 times more likely to have used a net (95% CI 1.3–6.0) compared to women with less education

**Conclusion:**

Distribution of ITNs in antenatal clinics in this setting is needed and feasible. The potential for ITN use by this target population is high.

## Background

There are between 300 and 500 million malaria infections and 1 million malaria attributed deaths worldwide each year [1]. The global malaria burden is not evenly distributed with Sub-Saharan Africa accounting for 90% of global malaria cases [2], and a majority of these cases occurring among women and children[3]. Malaria adversely impacts maternal health causing malaria-related anemia, and accounts for 20% of all childhood deaths in Africa [1,2]. Because of this, pregnant women and children under 5 have been targeted as key groups for malaria prevention. One major aim of the Roll Back Malaria (RBM) campaign is to have 80% of pregnant women and children under 5 covered by insecticide treated bed nets (ITN) by 2010[4].

Insecticide treated bed nets (ITNs) reduce human contact with mosquitoes and are an effective malaria prevention intervention[5]. ITNs have been shown to reduce severe disease and mortality due to malaria in endemic regions and reduce all cause mortality by approximately 20%[6]. Studies examining ITN effectiveness suggest a reduction in malaria episodes by 48 to 50% [7]. If used universally, ITNs could prevent approximately seven percent of the global under five mortality [8]. In addition to the direct benefit to the individual, ITN use offers an indirect, protective benefit for the community at large. Increased ITN use throughout a community has a more powerful impact than individual change[9].

Despite the knowledge that ITNs are effective in preventing malaria, there are known barriers to bed net ownership and use. Research suggests mosquito nuisance, perceived malaria risk, malaria knowledge, and socio-demographic factors, including education and household income, are important determinants of bed net ownership and use [10-12]. Studies show that those with more malaria knowledge and higher education and socioeconomic status are more likely to own a bed net [11-13]; however, knowledge of the benefits of bed nets and net ownership does not necessarily imply use. In a study of community factors relevant for participation in malaria control programs in Kenya, 88% of study respondents knew that bed nets protected from malaria, but only 55% actually used them [11]. Other important determinants of net ownership and use include seasonality, age, household size, and expenditure on other malaria prevention products [12-16].

In many African countries, ITN coverage is relative low (less than 10 percent in some areas) [12,17], however, coverage and use is increasing in the wake of the RBM and Global Fund efforts. ITN distribution practices vary ranging from sale to free distribution. Even in areas where ITNs are subsidized, bed nets remain out of reach for those who are the poorest and often at highest risk for malaria [18]. Several studies examining different net distribution practices have found providing nets free of charge to pregnant women to be an effective, low-cost malaria prevention strategy [9,19]. Supporters of ITN distribution believe that they should be treated as public goods and provided free of charge to affected communities [9]. However, free distribution of ITNs is not universally supported and in our study setting was opposed by some local stakeholders at the start of the study [20,21].

The data presented here is part of a larger study which aimed to determine whether ITNs distributed free of charge to pregnant women at their first antenatal clinic visit at the Bomoi maternity in Kinshasa were used from the time of distribution to delivery and in the six months following delivery. In this paper we describe socio-demographic characteristics, knowledge of malaria, ownership and use of bed nets and attitudes and norms surrounding bed net use among pregnant women at their baseline visit. We also explore factors associated with net ownership and use in this population at baseline.

### Study Setting

This study was conducted at the Bomoi maternity in Kinshasa, DRC which is managed by the Salvation Army. At Bomoi, approximately 250 new ANC patients are seen per month. In 2004, Bomoi clinic registers showed that approximately 149–200 deliveries occurred per month. A review of 47 ANC records at Bomoi conducted in January of 2005 found that 28% of women came for their first ANC visit at 6 months gestation, 25% of women came at 7 months and 25% at 8 months, 17% came at 5 months and about 4% came at less than 5 months (unpublished data). According to the 2007 Demographic and Health Surveys, 95.7% of pregnant women in Kinshasa had ANC by qualified staff [23]. As part of our efforts to strengthen prevention of mother to child transmission of HIV in antenatal clinics in Kinshasa, bed nets were going to be distributed at Bomoi as part of routine ANC services. The Bomoi medical management and staff were interested in conducting research to examine use of the nets if distributed free of charge.

HIV prevalence among women attending clinics for ANC in Kinshasa was approximately 2% between 2003–2005 [22]. Malaria is endemic in the DRC and transmission is predominantly continuous with seasonal fluctuations in intensity [24]. Malaria remains one of the primary causes of mortality and morbidity in the country, especially among pregnant women and young children[24].

## Methods

Beginning in November of 2005, long lasting, ITNs (Permanet 2.0) were distributed to pregnant women attending the Bomoi maternity as part of a prevention program of vertical transmission of HIV in Kinshasa, DRC. In order to assess the use of the ITNs provided free of charge, pregnant women attending their first antenatal care (ANC) visit were screened and, if eligible, enrolled in a cohort study. ITNs were provided free of charge to all pregnant women at their first ANC visit. As part of the study, women consented to be interviewed prior to receiving the net (baseline), at delivery and 6 months post-delivery. Women who were at least 18 years old who attended the Bomoi maternity for their first ANC visit were eligible to participate in the study. Women who attended their first ANC visit less than one month prior to their expected delivery date were not eligible to participate in the study. Eligible women were invited to draw a number to determine whether they were selected to take part in the study, 362 women were randomly selected. Women who provided informed consent to participate were given an appointment to complete the baseline interview; ITNs were only given after the baseline interview was completed. All women attending the maternity for ANC services, regardless of whether they enrolled in the study, received one ITN, material to hang the ITN, an educational leaflet that demonstrated how to properly hang the net, and a brief educational session on effective malaria prevention (including use of ITNs), transmission and symptoms of malaria.

Study participants were interviewed at the maternity in Lingala by trained interviewers using a pre-tested, structured questionnaire. The questionnaire covered socio-demographics; bed net ownership; information about individuals using bed nets; compliance with net use; knowledge and attitudes about malaria and bed net use, and perceived control over malaria prevention.

The primary outcomes of interest were bed net ownership and bed net usage at baseline. We also aimed to determine the frequency and distribution of socio-demographic characteristics and potential factors associated with bed net ownership and usage without distinguishing between insecticide treated and non-treated bed nets at baseline. Bivariate analyses were conducted to determine factors associated with net ownership and net usage using chi-square statistics. To identify factors we hypothesized to be associated with net ownership and net usage, we constructed separate multivariable logistic regression models. Based on a priori knowledge, we included socio-demographic variables in the model and then used backward elimination to identify other covariates. An alpha level of 0.10 was used to determine which variables remained in the model. Statistical analyses were conducted using SAS version 9.1.

## Results

Of the 362 women sampled to take part in the baseline questionnaire, 351 (97%) pregnant women were interviewed. Three women declined to participate; three were ineligible because they received an ITN before the baseline interview; two missed the baseline interview and were lost to follow-up, one missed the baseline interview but returned at delivery; one woman was found to be decisionally impaired; and one woman did not speak Lingala. Most women in this study were less than 35 years old, married, and had completed primary school (Table 1). About 72% of the women reported to have a working TV set in their home, 70% a radio, 25% reported to have a cell phone, and 23% a working refrigerator/freezer. Overall, 83% of the study population reported to own at least one durable item (Table 1).

**Table 1:** Demographics characteristics of women seeking antenatal care in Kinshasa, DRC in 2005/06.

	**N**	**%**
***Age***		
18–24 years	113	32.19
25–34 years	187	53.28
35 years and over	51	14.53
***Marital Status***		
Single	25	7.12
Married	277	78.92
Boyfriend/Partner	48	13.68
Separated	1	0.28
***Live with Partner***		
No	50	14.25
Yes	275	78.35
***First Pregnancy***		
No	238	67.81
Yes	113	32.19
***Highest Level of Education Attended***		
Completed Less than Primary School	25	7.12
Completed Primary School	8	2.28
Some Secondary School	208	59.26
Completed Secondary School	89	25.36
Completed University Education	17	4.84
Number of Durable Goods Owned*		
0	45	12.82
1	76	21.65
2	125	35.61
3	80	22.79
4	25	7.12

* Durable Goods include: cellular phone, radio, television, and fridge/freezer

Among 351 women interviewed at baseline, 115 (33%) already owned a bed net and 86 (25%) reported to have slept under the bed net the night before the interview. Almost half of the 236 women who did not own a bed net reported cost as a reason for not owning a net. When asked about reasons for not sleeping under a net, 87% of women stated not having a net as the reason (Table 2). Among net owners, over 50% slept under a net every night during the past week and approximately 70% slept under a net every night or almost every night in the past month (Table 2). Sixty-eight percent of all women reported having been diagnosed with malaria in the past year, of which 88% were self-diagnosed.

**Table 2:** Knowledge of malaria and use of bed net in antenatal clinic attendees in Kinshasa, DRC in 2005/06.

	N	%
***Causes of Malaria (unprompted)***		
Mosquitoes	317	90.31
Neighborhood Dirt	263	74.93
Standing Water	150	42.74
Other Answers*	62	17.66
***Do you know ways to prevent Malaria?***		
No	36	10.26
Yes	315	89.74
***Malaria diagnosis in the past year?***		
No	110	31.34
Yes	238	67.81
***Who diagnosed your Malaria?***		
Self diagnosed	210	88.24
Physician	28	11.76
***Number of People who Slept in the Home Last Night***		
0–3	133	37.89
4–6	116	33.05
7 or more	102	29.06
***Do you own a mosquito net?***		
No	236	67.24
Yes	115	32.76
Number of nets owned (mean, range)*§		1.4 (1–5)
***Reasons for not owning a mosquito net (unprompted)*^#^**		
Cost (Very Expensive)	114	48.31
Did not know about nets	27	11.44
Net was damaged	22	9.32
Not interested in a bed net	20	8.47
Do not like nets/Do not breathe well under net	13	5.51
No mosquitoes in my area	12	5.08
Other^†^	27	11.44
***Participant (pregnant woman) slept under a net last night***		
No	265	75.50
Yes	86	24.50
***Reasons for not sleeping under a net (unprompted)*^#^**		
Do not own a net	231	87.17
Mosquito net is torn	20	7.55
Other^††^	55	20.75
***Mother and child/children slept under a net last night (among those who slept under a net N = 100)***	27	27.8
***No one in home slept under a net last night***	251	71.51
***How many children under 5 slept under a net last night?*^§^**		
0	41	41.84
1	33	33.67
2 or more	24	24.49
***How many children ages 5–17 slept under a net last night?*^§^**		
0	62	63.92
1	17	17.53
2 or more	18	18.56
***Proportion of days slept under a net in the past week§***		
None	18	15.65
More than 0 but less than 100%	31	26.96
100%	66	57.39
***How often did you sleep under a mosquito net during the past month?§***		
Every night	52	45.22
Almost every night	29	25.22
A few nights	18	15.65
Not even once	16	13.91

* Includes: being in the rain, catching a cold, drinking dirty water, eating cold food, bugs/insects, suffocation/suppression at home, unclean or improperly washed bodies, trash/bad odors

† Includes: Do not know where to get a net, haven't bought a net yet, and Do not know (general)

†† Includes: Net causes other diseases, net not effective, chemical is dangerous for health, sleeping under a net is hot, spent the night outdoors b/c of heat, spent the night at a mourning, other family members used the net, mosquitoes can bite through the net, mosquitoes can get under the net, husband did not want us to sleep under it, no space in the room, other not specified, refused.

§Denominator includes only study participants who reported owning a mosquito net (N = 115).

# Participants were allowed to select more than one option

All participants reported they had heard of malaria. When asked about causes of malaria, 90% of the participants answered mosquitoes. Other common answers were neighborhood dirt and standing water (Table 2).

Attitudes and norms associated with malaria and bed net use are reported in Table 3. While only 25% reported sleeping under a net, over 98% of participants were worried about getting malaria, believed it is important and beneficial to sleep under a mosquito net every night, and believed sleeping under a mosquito net is a good way to protect themselves from malaria. However, less than 30% reported that obtaining a mosquito net in their community was easy. When comparing perceptions of free nets compared to nets that individuals would be required to buy, only 5% of participants felt that nets that one had to buy were more effective in preventing malaria compared to free nets and 14% reported they would prefer to use a net that they had bought themselves compared to one given to them for free. Over 80% of the women reported thinking that most people in their neighborhood would buy other things for their home if they had extra money, rather than buying a mosquito net.

**Table 3:** Attitudes and norms about malaria and bed net use among women seeking antenatal care in Kinshasa, DRC in 2005/06.

	Yes	
***In your opinion, do you think that most people where you live***	N	%
are worried/concerned about getting malaria	288	85.21
believe that using a mosquito net is the best way for prevention against malaria	298	87.65
would rather buy other things for home rather than buy a mosquito net	266	82.10
prefer using a mosquito net that they have bought rather than one given to them for free	86	29.76
***In your own opinion, do you believe***		
that you are worried about getting malaria	348	99.43
it is important and beneficial to sleep under a mosquito net every night	347	99.43
that sleeping under a mosquito net is a good way to protect yourself from malaria	346	98.86
you would prefer using a mosquito net you have bought better than one given to you for free	46	13.94
that you wouldn't afford to buy a mosquito net if you didn't receive one for free	55	15.80
it is useless to use a mosquito net because you can suffer from malaria anyway	77	22.13
that obtaining a mosquito net in the community where you live is easy	99	28.53
that malaria is a serious disease	341	97.15
that it is more difficult to use a mosquito net than taking drugs when you suffer from malaria	21	6.07
that children suffer from malaria more than adults do	265	79.10
it's difficult to use a mosquito net	28	8.07
that mosquito nets that you have to buy are better quality (more effective in preventing malaria) than nets that are given out for free	16	5.08

In bivariate analyses, married women were more likely to own a net (OR 2.7; 95% CI 1.4–5.1) and sleep under a net the night before the interview (OR 3.2; 95% CI 1.5–6.9) compared to single women (Table 4). Women who had completed secondary school education were significantly more likely to own and have slept under a net (OR 2.5 95% CI 1.6–4.1; OR 2.1 95% CI 1.2–3.4, respectively). Ownership of more durable goods was also significantly associated with net ownership and use (Table 4). Women who believed most people where they live were worried about getting malaria or who thought most people where they live preferred to use a net they had bought over one given to them for free were significantly less likely to own and have slept under a net (Table 5). Women who reported that they would prefer to use a net they had bought compared to one provided for free, who would not be able to afford a net if it was not provided for free, and who reported that children suffer from malaria more than adults were less likely to both own and sleep under a net (Table 5). Women who reported that obtaining a net in their community was easy were significantly more likely to own and have slept under a net.

**Table 4:** Bivariate analyses of factors associated with bed net ownership and sleeping under a mosquito net, among women seeking antenatal care in Kinshasa, DRC in 2005/06.

	**Net Ownership**		**Sleeping under a Net**	
**Variable**	**OR**	**95% CI**	**OR**	**95% CI**

***Age***				
18–24 years	0.81	0.49, 1.3	0.78	0.45, 1.3
25–34 years	1.0		1.0	
35 years and over	0.86	0.44, 1.7	0.75	0.36, 1.6
***Marital Status***				
Single	1.0		1.0	
Married	2.7	1.4, 5.1	3.2	1.5, 6.9
***Living with Partner***				
No	1.0		1.0	
Yes	1.2	0.51, 2.7	0.55	0.20, 1.5
*Ever Attended School*				
No	1.0		1.0	
Yes	2.1	0.29, 14	3.1	0.43, 23
***First Pregnancy***				
No				
Yes	0.94	0.58, 1.5	0.77	0.45, 1.3
***Highest Level of Education Completed***				
Less than Secondary School	1.0		1.0	
Secondary School or More	2.5	1.6, 4.1	2.1	1.2, 3.4
***Employment Status***				
Never Employed	1.0		1.0	
Not Currently Employed	1.5	0.85, 2.8	1.7	0.88, 3.1
Employed	0.86	0.52, 1.4	0.79	0.45, 1.4
***Number of Durable Goods Owned*^†^**				
0	1.0		1.0	
1	1.5	0.63, 3.7	1.4	0.55, 3.8
2	1.6	0.68, 3.6	1.4	0.54, 3.4
3	3.8	1.4, 7.7	3.3	1.3, 8.2
4	5.1	1.7, 14	2.6	0.80, 8.2
***Owns Durable Goods***				
No	1.0		1.0	
Yes	2.1	0.98, 4.6	1.9	0.81, 4.4
***Do you know ways to prevent Malaria?***				
No	1.0		1.0	
Yes	19	2.7, 146	27.5	1.7, 453
***Malaria diagnosis in the past year?***				
No	1.0		1.0	
Yes	1.2	0.79, 2.0	1.3	0.80, 2.2

**Table 5:** Bivariate analyses of factors associated with bed net ownership and use among women seeking antenatal care in Kinshasa, DRC in 2005/06.

	**Net Ownership**		**Sleeping under a Net**	
***In your opinion, do you think that most people where you live***	OR	95% CI	OR	95% CI
are worried/concerned about getting malaria	0.52	0.28, 0.95	0.52	0.27, 0.98
believe that using a mosquito net is the best way for prevention against malaria	0.84	0.43, 1.7	0.68	0.34, 1.4
would rather buy other things for home rather than buy a mosquito net	1.4	0.76, 2.7	1.6	0.76, 3.3
prefer using a mosquito net that they have bought rather than one given to them for free	0.47	0.26, 0.85	0.44	0.23, 0.86
				
***In your own opinion, do you believe*^‡^**				
you would prefer using a mosquito net you have bought better than one given to you for free	0.43	0.26, 0.69	0.32	0.18, 0.58
you wouldn't afford to buy a mosquito net if you didn't receive one for free	0.30	0.13, 0.65	0.33	0.13, 0.79
it is useless to use a mosquito net because you can suffer from malaria anyway	0.79	0.45, 1.4	1.1	0.62, 2.0
that obtaining a mosquito net in the community where you live is easy	1.7	1.0, 2.7	1.8	1.1, 3.1
that it is more difficult to use a mosquito net than taking drugs when you suffer from malaria	0.64	0.23, 1.8	1.3	0.48, 3.4
that children suffer from malaria more than adults do	0.39	0.23, 0.66	0.41	0.23, 0.72
it's difficult to use a mosquito net	0.41	0.15, 1.1	0.48	0.16, 1.4
that mosquito nets that you have to buy are better quality (more effective in preventing malaria) than nets that are given out for free	1.3	0.45, 3.8	1.1	0.35, 3.6

* OR = Odds Ratio; 95% CI = 95% Confidence Interval

‡ The following variables were excluded from the bivariate analysis due to lack of variance in the response categories: worry about getting malaria, belief that sleeping under a net is beneficial, belief that sleeping under a net is good protection from malaria, and belief that malaria is a serious disease.

In multivariable analyses examining factors associated with net ownership and use, education remained strongly associated with both. Women who had secondary school or higher education were 3.4 times more likely to own a net and 2.8 times more likely to have used a net compared to women with less than secondary school education (Table 6 and 7). Although not statistically significant, women who reported owning more durable goods were more likely to own a bednet than women owning no durable goods. In addition, married women were also more likely to own nets than non-married women (OR 2.5 95% CI 0.74–8.8). Attitudes around bednet use were also important. Women who reported thinking that mosquito nets that are bought are better quality than ones given out for free were less likely to own a net (OR 0.36 95% CI 0.15–0.88). Women who reported that they thought mosquito nets were difficult to use or that children suffer from malaria more than adults were also less likely to own nets; these associations were not statistically significant (Table 6).

**Table 6:** Adjusted Odds Ratios (OR) and 95% Confidence Intervals (CI) examining factors associated with net ownership in multivariable analyses among women seeking antenatal care in Kinshasa, DRC in 2005/06.

**Variable**	**OR**	**95% CI**
***Age***		
17–24	1.2	0.49, 3.1
25–34	1.0	
35 and over	0.84	0.29, 2.4
***First Pregnancy***		
No	1.0	
Yes	0.75	0.29, 1.9
***Highest Level of Education Completed***		
Less than Secondary School	1.0	
Secondary School or Beyond	3.4*	1.6, 7.3
*Number of Durable Goods Owned*		
0	1.0	
1–2	2.2	0.60, 7.8
3–4	3.1	0.80, 11
***Employment Status***		
Never had a job	1.0	
Currently unemployed	1.1	0.41, 2.8
Currently employed	0.62	0.28, 1.4
***Living with Partner***		
No	1.0	
Yes	0.70	0.26, 1.9
***Marital Status***		
Single	1.0	
Married	2.5	0.74, 8.8
***Believe mosquito nets that you have to buy are better quality (more effective in preventing malaria) than nets that are given out for free***		
No	1.0	
Yes	0.36*	0.15, 0.88
***Believe children suffer from malaria more than adults***		
No	1.0	
Yes	0.49	0.22, 1.1
***Think mosquito nets are difficult to use***		
No	1.0	
Yes	0.19	0.034, 1.0

* Statistically significant at the alpha = 0.05 level

† Durable Goods include: cellular phone, radio, television, and fridge/freezer

**Table 7:** Adjusted Odds Ratios (OR) and 95% Confidence Intervals (CI) examining factors associated with sleeping under a net the night before the interview in multivariable analyses among women seeking antenatal care in Kinshasa, DRC in 2005/06.

Variable	OR	95% CI
***Age***		
17–24	1.4	0.57, 3.6
25–34	1.0	
35 and over	0.95	0.31, 2.9
***First Pregnancy***		
No	1.0	
Yes	0.66	0.26, 1.7
***Highest Level of Education Completed***		
Less than Secondary School	1.0	
Secondary School or Beyond	2.8*	1.3, 6.0
***Number of Durable Goods Owned***		
0	1.0	
1–2	1.1	0.32, 3.9
3–4	1.7	0.46, 6.3
***Employment Status***		
Never had a job	1.0	
Currently unemployed	0.91	0.35, 2.4
Currently employed	0.55	0.23,1.3
*Living with Partner*		
No	1.0	
Yes	1.2	0.41, 37
***Marital Status***		
Single	1.0	
Married	1.5	0.43, 5.6
***Believe children suffer from malaria more than adults***		
No	1.0	
Yes	0.45	0.20, 1.0

* Statistically significant at the alpha = 0.05 level

† Durable Goods include: cellular phone, radio, television, and fridge/freezer

## Discussion and conclusion

In this population of pregnant women attending their first antenatal clinic visit in a poor, urban neighborhood of Kinshasa, one third of women reported owning a bed net and one quarter reported having slept under a bed net the night before the interview. These numbers are higher than we anticipated, particularly given the poor environment in which these women live. However, given that malaria is endemic in Kinshasa and that close to 70% of women reported having malaria in the past year, the coverage reported in this population is certainly far from meeting the 80% goal set by the Roll Back Malaria (RBM) Partnership for the year 2010. At the time of this study, bed nets were not widely available in Kinshasa and were primarily available through social marketing and commercial outlets. Shortly after the start of this study, ITNs began to be distributed through organizations in the DRC working in partnership with the Global Fund to fight AIDS, Tuberculosis and Malaria (Global Fund). Use of ITNs among pregnant women has been found to be increasing in many malaria endemic parts of Africa, particularly in areas where the Roll Back Malaria campaign and Global Fund are active. In Ghana in 2000, 29% of nursing mothers/pregnant women reported sleeping under an ITN the night before the survey, this increased to 58% in 2003 [25]. In a community survey of six African countries, use of ITNs in 2004 by pregnant women of reproductive age varied from a low of 32% to a high of 69% [26]. In another survey in Burkina Faso where pregnant women were interviewed in ANC clinics and Delivery Units, 58% of women reported owning an ITN in 2004 [27].

The majority of women in this study reported that the reason they did not sleep under an ITN was because they did not own one. Lack of affordability was reported as an important barrier to ITN ownership. Cost has been reported as a major barrier to ITN ownership and use in other studies [28-30]. This study was undertaken because several local stakeholders opposed the free distribution of bednets believing that selling ITNs at a subsidized price promotes better use of ITNs and it helps to cover some of the distribution costs which promote sustainability of net distribution. Contrary to beliefs of local stakeholders, the vast majority of women in this study did not report thinking that bednets provided free of charge were less effective or that they would prefer to use one that they had bought compared with one provided free of charge. Two recent studies have found that free-distribution of ITNs has resulted in substantial increases in net coverage compared to subsidized, "social marketing" approaches[31,32]. Currently, the maternity center where this study was conducted sells ITNs at a highly subsidized price in the community surrounding the clinic; in principle, indigent persons can receive bed nets free of charge. It is likely that distribution strategies that utilize a range of approaches, both subsidized sales as well as free distribution, will be most successful in reaching mass coverage[21]. However, mass, free distribution might be necessary to achieve the high targets for ITN coverage in high risk population set by RBM and others[33].

The majority (69%) of women in this study population had less than a secondary school education and 64% were unemployed. However, the overall understanding of malaria transmission and effective means to prevent transmission were good in this population. Women reported being worried about contracting malaria and reported positive attitudes towards using bed nets to prevent malaria. In multivariable analyses, one of the few factors that was strongly associated with net ownership and net use was having secondary school education or higher. Increased education, particularly for young women, has been found to be associated with a number of beneficial health outcomes, including reduced infant and maternal mortality[34]. With regard to bed net use, other studies in Africa have found that women with more education were more likely to own and use bed nets [28,29].

### Strengths and Limitations

A major strength of this study is its longitudinal nature which will allow us to examine the relationship between baseline knowledge and attitudes to bed net usage in the future. In addition, the study includes participants' opinions about their preferences for free compared to purchased bed nets; this information has not been included in other studies on this topic. The results from this study are consistent with the findings of other studies, indicating the validity of the results obtained from this analysis.

We acknowledge the limitations of the study sample and that it may not be representative of all women attending ANC in Kinshasa and certainly not of all pregnant women in Kinshasa. We do not attempt to generalize to all women attending ANC. However, these women are unlikely to be very different from many women seeking ANC in Kinshasa. The study is also limited in that we only sampled from a single clinic. We selected one facility that was considered typical of many ANC facilities in poor communities in Kinshasa that have a high malaria burden. Research resources did not permit city-wide sampling. This is a clinic-based sample, and women come from the Bomoi clinic's catchment area. We did not collect women's addresses but given the high transportation costs in Kinshasa and the low purchasing power of the population it is unlikely that the women came from other neighborhoods. Another limitation is that we did not quantify the number or types of household members (for example, under 5, 5–17, etc.) therefore we cannot report on the proportion of each age group that slept under bed nets, we can only report if anyone in a particular category slept under a net. In addition, all the data collected in this study were self-reported and therefore subject to recall bias. We do not anticipate recall will significantly bias the results since a majority of the questions ask about bed net usage the night before the interview.

Free distribution of ITNs in antenatal clinics to pregnant women seems acceptable in this setting. The potential for ITN use by this target population is high. Distribution of ITNs through antenatal care clinics, particularly as part of vertical HIV transmission prevention programs, in malaria endemic areas may be a highly efficient way to increase access and use of ITNs during a time when women are more vulnerable to malaria. Use of ITNs distributed free of charge in this population will be evaluated among these women at delivery and among a sub-set of women 6 months post delivery.

## Competing interests

The authors declare that they have no competing interests.

## Authors' contributions

AP was involved in the design, implementation, analysis, interpretation and write up of this study. ET was involved in the analysis and write up of this study. DN was involved in the design and implementation of this study and in editing the main paper. SD was involved in the implementation, interpretation and write up of the study. MT was involved in the implementation of this study and in editing the main paper. KM was involved in analysis and interpretation of the study findings. SM was involved in the design of the study, the interpretation of study findings and editing of the paper FB was involved in the design and implementation of the study, the interpretation of study findings and write up of the study. All authors read and approved the final manuscript.

## Pre-publication history

The pre-publication history for this paper can be accessed here:


